# CSF Levels of Elongation Factor Tu Is Associated With Increased Mortality in Malawian Adults With *Streptococcus pneumoniae* Meningitis

**DOI:** 10.3389/fcimb.2020.603623

**Published:** 2020-12-11

**Authors:** Emma C. Wall, Philip Brownridge, Gavin Laing, Vanessa S. Terra, Veronica Mlozowa, Brigitte Denis, Mulinda Nyirenda, Theresa Allain, Elisa Ramos-Sevillano, Enitan Carrol, Andrea Collins, Stephen B. Gordon, David G. Lalloo, Brendan Wren, Robert Beynon, Robert S. Heyderman, Jeremy S. Brown

**Affiliations:** ^1^The Francis Crick Institute, London, United Kingdom; ^2^Division of Infection and Immunity, University College London, London, United Kingdom; ^3^Malawi-Liverpool-Wellcome Trust Clinical Research Programme, College of Medicine, University of Malawi, Blantyre, Malawi; ^4^Centre for Proteomics, Institute of Integrative Biology, University of Liverpool, Liverpool, United Kingdom; ^5^Liverpool School of Tropical Medicine, Liverpool, United Kingdom; ^6^London School of Hygiene and Tropical Medicine, London, United Kingdom; ^7^Adult Emergency Trauma Centre, Queen Elizabeth Central Hospital, Ministry of Health, Blantyre, Malawi; ^8^College of Medicine, University of Malawi, Blantyre, Malawi; ^9^UCL Respiratory, Division of Medicine, University College London, London, United Kingdom; ^10^Institute of Infection and Global Health, University of Liverpool, Liverpool, United Kingdom; ^11^Liverpool University Hospital Foundation Trust, Liverpool, United Kingdom

**Keywords:** meningitis, HIV—human immunodeficiency virus, mortality, *Streptococcus pneumoniae*, cerebrospinal fluid, proteomics, Elongation factor Tu (EF-Tu)

## Abstract

**Background:**

Mortality from bacterial meningitis, predominately caused by *Streptococcus pneumoniae*, exceeds 50% in sub-Saharan African countries with high HIV prevalence. Underlying causes of high mortality are poorly understood. We examined the host and pathogen proteome in the CSF of adults with proven pneumococcal meningitis (PM), testing if there was an association between differentially expressed proteins and outcome.

**Materials/Methods:**

CSF proteomes were analyzed by quantitative Mass-Spectrometry. Spectra were identified using the Swissprot human and TIGR4 pneumococcal protein libraries. Proteins were quantitated and analyzed against mortality. Unique proteins in PM were identified against published normal CSF proteome. Random-Forest models were used to test for protein signatures discriminating outcome. Proteins of interest were tested for their effects on growth and neutrophil opsonophagocytic killing of *S. pneumoniae*.

**Results:**

CSF proteomes were available for 57 Adults with PM (median age 32 years, 60% male, 70% HIV-1 co-infected, mortality 63%). Three hundred sixty individual human and 23 pneumococcal proteins were identified. Of the human protein hits, 30% were not expressed in normal CSF, and these were strongly associated with inflammation and primarily related to neutrophil activity. No human protein signature predicted outcome. However, expression of the essential *S. pneumoniae* protein Elongation Factor Tu (EF-Tu) was significantly increased in CSF of non-survivors [False Discovery Rate (q) <0.001]. Expression of EF-Tu was negatively co*-*correlated against expression of Neutrophil defensin (r 0.4 p p < 0.002), but not against complement proteins C3 or Factor H. *In vitro*, addition of EF-Tu protein impaired *S. pneumoniae* neutrophil killing in CSF.

**Conclusions:**

Excessive *S. pneumoniae* EF-Tu protein in CSF was associated with reduced survival in meningitis in a high HIV prevalence population. We show EF-Tu may inhibit neutrophil mediated killing of *S. pneumoniae* in CSF. Further mechanistic work is required to better understand how *S. pneumoniae* avoids essential innate immune responses during PM through production of excess EF-Tu.

## Introduction

Acute bacterial meningitis (ABM) is a leading cause of infectious mortality and morbidity worldwide; an estimated 2.8 million incident cases of community-acquired ABM were reported in 2016 occurring predominately in children and young people (Collaborators GBDM, 2018). There is a particularly high toll of meningitis caused by *Streptococcus pneumoniae* (pneumococcal meningitis, PM) in sub-Saharan Africa, where the combination of high HIV prevalence and high burden of nasopharyngeal carriage create a potent environment for PM to flourish in all age groups (Gessner et al., 2010; Heinsbroek et al., 2015; Britz et al., 2016; Swarthout et al., 2020). Ambitious global WHO targets to defeat meningitis by 2030 were published in 2018 (Organisation) WWH, [[NoYear]]). However, progress in Africa is limited by the lack of affordable vaccines and effective adjunctive therapies to antibiotics (Scarborough et al., 2007; Ajdukiewicz et al., 2011; Wall et al., 2017a). In African LMICs mortality from ABM in adults and adolescents exceeds 50% compared to 10–20% in better resourced settings, but causes of excessive mortality from ABM in this setting are not well described (van de Beek et al., 2010; Mourvillier et al., 2013; Wall et al., 2017a; Tenforde et al., 2019).

Prognostic scores for ABM have low sensitivity and specificity (Weisfelt et al., 2008; Wall et al., 2017b), suggesting pathological differences in the CNS leading to poor outcome are not readily detected by clinical parameters. During PM, large numbers of neutrophils rapidly trans-migrate from blood in response to pro-inflammatory mediators in CSF (Potter and Harding, 2001; Koedel et al., 2009; de Oliveira et al., 2016). Neutrophils have a critical role in killing *S. pneumoniae* by phagocytosis (Ramos-Sevillano et al., 2016; Ullah et al., 2017), but also contribute to counter-productive inflammatory responses which may mediate death and disability in pneumococcal meningitis, sepsis and pneumonia (Bewley et al., 2011; Ramos-Sevillano et al., 2016; Ritchie et al., 2018; Domon et al., 2018). This host-pathogen interaction triggers an inflammatory cascade of both cytotoxic effects of host pro-inflammatory mediators (Mook-Kanamori et al., 2011; Wang et al., 2016), and bacterial toxins, that drive tissue damage in non-survivors characterized by apoptotic neuronal cell injury, raised intracranial pressure (ICP), thrombosis, cerebral edema, and ischemia (Wall et al., 2012; Wippel et al., 2013; Wall et al., 2014; Doran et al., 2016).

Proteomics provides an opportunity to dissect this host-pathogen interaction in CSF during disease by both quantitating the relative abundance of multiple inflammatory proteins, and testing for associations between human and bacterial proteins and outcome (Zhang et al., 2015; Bastos et al., 2017). Previously, the CSF proteome in a small number of children with PM from our center showed marked upregulation of multiple inflammatory and bacterial proteins compared to hospital controls, including neutrophil proteins S100A9 and myeloperoxidase in CSF (Gomez-Baena et al., 2017). In an earlier study, using 2D electrophoresis proteomics of adults with PM, demonstrated consumption of complement C3 in non-survivor CSF, we described an exacerbated host response including proteins involved with brain damage (Goonetilleke et al., 2010; Goonetilleke et al., 2012), but did not find major proteomic differences between the outcome groups.

In this study, we utilized label-free quantitative tandem mass-spectrometry proteomics to quantitate the host and pathogen proteome in adults with PM, to determine if a CSF protein signature predicts the outcome from PM. We further tested the effects of a protein associated with poor outcome using an *in vitro* neutrophil killing assay.

## Methods

### Patients

CSF was obtained for proteomics from adults and adolescents on admission to hospital with suspected bacterial meningitis at Queen Elizabeth Central Hospital in Blantyre, Malawi between 2011 and 2013 (Wall et al., 2017a). All CSF samples were collected prior to administration of parenteral antibiotics 2 g BD for 10 days (Scarborough et al., 2007; Ajdukiewicz et al., 2011). Clinical data were recorded on admission to hospital, clinical outcome data reported at 6 weeks post-discharge (Wall et al., 2017a).

### Procedures

Routine CSF microscopy, cell count, and CSF culture was done at the Malawi-Liverpool-Wellcome Trust Clinical Research Programme laboratory in Blantyre, Malawi as previously described (Wall et al., 2017a). Culture negative samples were screened using the multiplex real-time polymerase chain reaction for *S. pneumoniae*, *Neisseria. Meningitidis*, and *Haemophilus influenzae type b* (Hib) kit from Fast-Track Diagnostics (FTD Luxemburg) according to the manufacturer’s instructions, bacterial loads were estimated from Ct values. Additionally, CSF was screened for Herpes viruses including EBV, CMV, and HSV1. We excluded patients with active viral co-infection in the CSF. We collected 2.0 ml of CSF for proteomics, stored on receipt in the laboratory (within 2 h of LP) at −80 degrees Celsius. In-hospital HIV testing was done on all patients by the clinical teams using point-of care Genie™ HIV1&2 test kits (BioRad, USA).

### CSF Protein Extraction and Mass-Spectrometry

Protein concentration in all CSF samples was measured by nanodrop (Thermo Scientific, UK) and normalized to 200 µg/ml. Samples were centrifuged at 13,000 *g* and the pellet stored at −80°C until peptide extraction. Proteins were treated with the surfactant 0.1% (v/v) RapiGest™ (Waters) at 80°C for 10 min followed by reduction with diothiothreitol (DTT) at a final concentration of 3 mM (60°C for 10 min) and alkylation with iodoacetamide (IAA) at a final concentration of 50 mM (room temp, in the dark, 1 h). The enzyme trypsin (sequencing grade, Promega) was added at an enzyme:substrate ratio of 1:50 and incubated overnight with agitation at 37°C. The surfactant was inactivated the following day by treatment with 0.1% trifluoroacetic acid (TFA) (37°C for 1 h) and peptides were recovered following centrifugation at 13,000 *g*.

Resultant CSF peptides were separated by RPLC using a DIONEX UltiMate™ 3000LC chromatography system and MSMS analysis performed on an LTQ Orbitrap Velos using Xcalibur software v2.1 (both Thermo Scientific, UK). Peptides (10 µl = ~500 ng) were injected onto the analytical column (Dionex Acclaim® PepMap RSLC C18, 2 µm, 100 Å, 75 µm i.d. ×15 cm, nanoViper.), which was maintained at 35°C and at a nanoflow rate of 0.3 µlmin^−1^. Peptides were separated over linear chromatographic gradients composed of buffer A (2.5% ACN: 0.1% FA) and buffer B (90% ACN: 0.1% FA). Two gradients, 60 (3–50% buffer B in 40 min) and 180 min (3–60% buffer B in 140 min), were employed for analysis. Full scan MS spectra were acquired over the *m/z* range of 350–2,000 in positive polarity mode by the Orbitrap at a resolution of 30,000. A data-dependent Top20 collision induced dissociation (CID) data acquisition method was used. The ion-trap operated with CID MSMS on the 20 most intense ions (above the minimum MS signal threshold of 500 counts).

### Bio-informatic Quantitative Analysis

Data was initially mass recalibrated using the mzRefiner filter of the Proteowizard msconvert tool. The resulting files were then processed using Progenesis QI (version 2 Nonlinear Dynamics, Newcastle upon Tyne, UK). Samples were aligned according to retention time using a combination of manual and automatic alignment. Default peak picking parameters were applied and features with charges from 1+ to 4+ featuring three or more isotope peaks were retained. Database searching was performed using Mascot (Matrix Science, London, UK). A Mascot Generic File, created by Progenesis QI, was searched against a merged database of the reviewed entries of the Uniprot reference proteome set of *H. sapiens* (09/12/2015, 20,187 sequences) and *S. pneumoniae* (09/12/2015, 2,030 sequences). A fixed carbamidomethyl modification for cysteine and variable oxidation modification for methionine were specified. A precursor mass tolerance of 10 ppm and a fragment ion mass tolerance of 0.6 Da were applied. The results were then filtered to obtain a peptide false discovery rate of 1%. Protein inference and quantification was performed using the “Relative Quantitation using non-conflicting peptides” option in Progenesis. Protein quantification values are determined from all peptides but weighted according to peptide intensity. Proteins were annotated as differentially expressed if they achieved an FDR corrected q value of 0.05. Outcome prediction was performed by random forest using the cforest function of the “party” package in R. Pathways analysis of proteins was done using Innate DB.

### Synthesis of Pneumococcal Proteins

*E. coli* cells containing pEQ30_EF-Tu were kindly donated by Prof. Sven Hammerschmidt. *E. coli* was grown at 25°C in LB supplemented with 100 µg/ml ampicillin (Mohan et al., 2014). When the OD_595nm_ reached 0.5, protein expression was induced by adding 1 mM isopropyl β-d-thiogalactoside (IPTG). EF-Tu was modified to contain a polyhistidine tag to aid purification and detection. EF-tu is insoluble when expressed in *E. coli* and precipitates in inclusion bodies. Firstly, the cells were pelleted by centrifugation at 3,250 × g, then the pellet was resuspended in 10 ml of 50 mM NaH_2_PO_4_, 300 mM NaCl (pH 8). This was followed firstly by sonication and then by centrifugation at 4,300 × g, for 30 min at 4°C. The pellet containing the inclusion bodies was then washed in 50 mM NaH_2_PO_4_, 300 mM NaCl (pH 8) and resuspended in 500 µl of 50 mM NaH_2_PO_4_, 300 mM NaCl, 4 M Urea (pH 8) to solubilize the inclusion bodies. This suspension was then centrifuged at 18,000 × g for 30 min at 4°C. The supernatant that contained EF-Tuf was then mixed with Ni-NTA (Qiagen, Germany) that had been resuspended in the same buffer as the protein. The column was then washed with 10 column volumes (CV) of 50 mM NaH_2_PO_4_, 300 mM NaCl, 4 M Urea (pH 8). The protein was eluted with 6 CV’s using 50 mM NaH_2_PO_4_, 300 mM NaCl, 4 M Urea, 250 mM immidazole (pH 8). Eluted EF-Tuf was refolded by dialysis in 20 mM Tris-HCl 5 mM MgCl2 pH 7.4. Finally, the protein was recovered, and the buffer exchanged into PBS during concentration. Presence of the protein was confirmed *via* an anti-His western blot. Identity of the protein of choice was confirmed by running an anti-His western blot of the cell lysates prepared in the presence and absence of IPTG. Non-induced cell extracts did not react with anti-his antibody (**Supplementary Figure 1**).

### Bacterial Growth Conditions

Strains of *S. pneumoniae* serotype 1 ST5316 (Terra et al., 2020) were grown in Todd-Hewitt broth supplemented with 0.5% yeast extract (THY) to OD 0.5 and stored in 80% glycerol at −80°C as previously described (Ramos-Sevillano et al., 2012). Bacteria were thawed, washed in PBS twice, and diluted to 1 × 10^6^ CFU/ml in either pooled serum from five healthy laboratory donors (non-PCV vaccinated) or thawed human cerebrospinal fluid (CSF). CSF was obtained from patients who underwent therapeutic lumbar puncture for idiopathic intracranial hypertension, all had biochemically normal, acellular CSF. CSF was kindly donated by Professor Diederik van de Beek (Amsterdam Medical Centre, Netherlands). Four ug of purified EF-Tu, bovine serum albumin (BSA), NanA or PiaA or PBS control were added to five technical replicates.

Growth for 24 h was detected by changes in optical density at 620 nm with shaking in a microplate reader (Tecan® USA) at 37°C.

### Opsonophagocytosis Assays

Bacterial killing by purified human neutrophils was done using a previously described opsonophagocytosis assay (Hyams et al., 2010). Briefly, neutrophils were extracted from whole blood of healthy donors by negative selection Maxspress kit (Milteyni biotech, USA) according to the manufacturer’s instructions. Bacteria were opsonized in either 10% pooled serum or CSF for 30 min at 37°C then incubated with purified fresh human neutrophils in HBSS with either 4 ug of purified EF-Tu, BSA, or PBS at 37°C for 45 min with shaking in the dark. Cytochalasin D was included in the negative control to stop phagocytosis. Serial dilutions of the reaction mix were plated on Colombia agar supplemented with 5% horse blood, and colony forming units (CFU) counted after 18 h incubation at 37°C.

### Statistical Methods

All conventional statistical tests were two tailed, alpha <0.05 determined statistical significance. Ninety-five percent confidence intervals are presented for odds ratios. Logistic regression was used to model associations between clinical outcomes and risk factors while controlling for confounding factors.

### Data Deposition

All proteomic data was submitted to the ProteomeXchange: Accession number:

Submission Reference: 1-20200901-167440

Submission Path:/nfs/pride/drop/pride-drop-003/philipjb_20200901_134730

### Ethical Approvals

All participants or nominated guardians gave written informed consent for inclusion. Ethical approval for the transcriptomics study was granted by both the College of Medicine Research and Ethics Committee (COMREC), University of Malawi (P.01/10/980, January 2011), and the Liverpool School of Tropical Medicine Research Ethics Committee, UK (P10.70, November 2010) Committee, Liverpool, UK.

## Results

### Patients

Fifty-seven patients with proven PM, whose CSF was stored within 4 h of presentation to hospital, were included in the study, 33 (57%) of whom had died within 6 weeks (**Table 1**). Median age was 33 years (IQR 26–44), and predominantly HIV co-infected (34/47, 72%). CSF protein and bacterial loads were high, and although raised, the CSF white cell counts (WCC) were substantially lower than those reported in high income settings (Costerus et al., 2016) (**Table 1**). Neither CSF bacterial load, CSF protein, nor CSF WCC differed significantly between survivors and non-survivors in an un-adjusted analysis. Similarly, to our previous reports, Glasgow Coma Score (GCS) was significantly lower in non-survivors (11/15) than survivors (14/15) (OR 0.57, 95% CI 0.35–0.87) on admission (Wall et al., 2017b).

**Table 1:** Demographic details of included participants.

	Survivors (n = 19)	Non-survivors (n = 33)	OR Survival	p
**Bacterial load (log_10_)**	4.7 × 10^6^ (7.7 × 10^5^–3.5 × 10^7^)	1.4 × 10^7^ (9.8 × 10^5^–6.8 × 10^7^)	1.1 (0.83 : 1.56)	0.5
**Age (years)**	30 (26–41)	36 (24–44)	1.02 (0.96 : 1.06)	0.3
**Male gender**	11 (58%)	21 (63%)	0.78 (0.24 : 2.4)	0.68
**GCS**	14 (13–15)	12 (8–13)	0.57 (0.35 : 0.85)	0.007
**CSF WCC cells/mm^3^**	52 (2–1760)	27 (2–287)	0.83 (0.43 : 1.5)	0.56
**CSF protein (g/dl)**	3.02 (2.5–4.0)	2.52 (1.8–5.6)	1.1 (0.7 : 1.7)	0.63
**HIV infection**	10/16 (62%)	24/31 (77%)	0.48 (0.1 : 1.8)	0.28

### CSF Human Proteome

**Figure 1:**
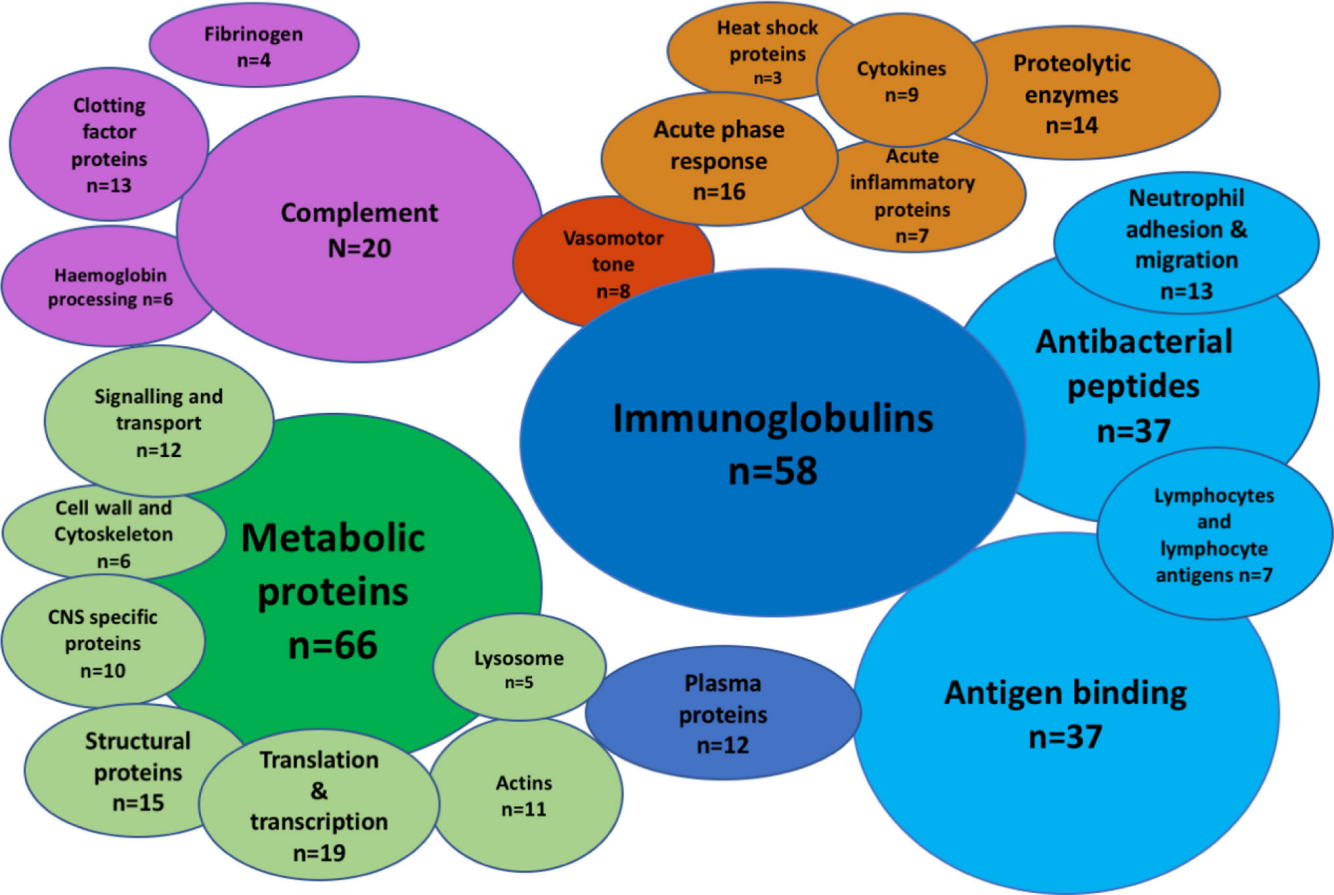
The CSF proteome in pneumococcal meningitis is diverse and highly inflammatory. Bubble plot summary of the CSF proteome in pneumococcal meningitis. Three hundred thirty-six peptides identified by Mass-Spectrometry. Green, cellular functional proteins; blue, proteins synthesized by white and plasma cells; purple, complement and clotting cascades; orange, pro-inflammatory proteins.

A total of 336 peptides matching Uniprot identifiers were found in the CSF (**Figure 1**, **Supplementary Data 1**). Albumin was the most abundant CSF protein, followed by sub-classes of immunoglobulins, complement C3, alpha-1 antitrypsin, haptoglobin, hemopexin, and neutrophil proteins S100A8&9 (**Table 2**). Overall, the most abundant protein classes were immunoglobulins, metabolic proteins, and complement, along with antigen-binding proteins, neutrophil-associated and anti-bacterial proteins, inflammatory response, and vasomotor tone. Blood brain barrier (BBB) breakdown was suggested by the identification of hemoglobin, haptoglobin, and fibrinogen in the CSF (**Figure 1**). Pathways analysis of the entire proteome (Innate DB) found 16 pathways enriched with q < 0.05, dominated by complement, platelet degranulation, scavenging of heme, and axon guidance pathways (**Table 3**).

**Table 2:** Estimated abundance (arbitrary quantitative units) of 20 most highly expressed proteins in the CSF of patients with PM.

**#**	Protein	Median protein abundance (units)	#	Protein	Median protein abundance
**1**	Serum albumin	2.02E+09	**13**	Hemopexin	4.86E+07
**2**	Ig gamma-1 chain C region	1.39E+09	**14**	Apolipoprotein A-I	3.24E+07
**3**	Ig kappa chain C region	6.02E+08	**15**	Fibrinogen gamma chain	2.63E+07
**4**	Immunoglobulin lambda-like polypeptide 5	2.91E+08	**16**	Protein S100-A9	2.51E+07
**5**	Alpha-1-acid glycoprotein 1	1.33E+08	**17**	Alpha-2-macroglobulin	2.49E+07
**6**	Ig lambda-2 chain C	1.25E+08	**18**	Ig heavy chain V-III region BRO	2.46E+07
**7**	Haptoglobin	1.09E+08	**19**	Neutrophil defensin 1	2.25E+07
**8**	Alpha-1-antitrypsin	9.06E+07	**20**	Alpha-1-acid glycoprotein	2.11E+07
**9**	Ig alpha-1 chain C region	9.04E+07	**21**	Complement C3	2.10E+07
**10**	Serotransferrin	8.65E+07	**22**	Vitamin D-binding protein	2.09E+07
**11**	Ig gamma-3 chain C region	6.67E+07	**23**	Protein S100-A8	1.91E+07
**12**	Ig gamma-2 chain C region	6.39E+07	**24**	Ig mu heavy chain disease protein	1.88E+07

**Table 3:** Enrichment of 10 most highly expressed biological pathways in the CSF of patients with PM.

Pathway number	Pathway name	P-adj value enrichment
**1**	Innate immune system	3.23E-73
**2**	Immune system	3.72E-61
**3**	Complement cascade, Regulation of complement cascade	3.29E-59
**4**	Hemostasis	7.66E-53
**5**	Neutrophil degranulation	3.29E-43
**6**	Scavenging heme from plasma	4.16E-41
**7**	RHO GTPases activate PKNs (actin dynamics)	1.70E-39
**8**	Binding and Uptake of Ligands by Scavenger Receptors	2.77E-39
**9**	Classical antibody-mediated complement activation	1.23E-36
**10**	Platelet degranulation	7.48E-36

### Sub-Set of Proteins Unique to PM

**Figure 2:**
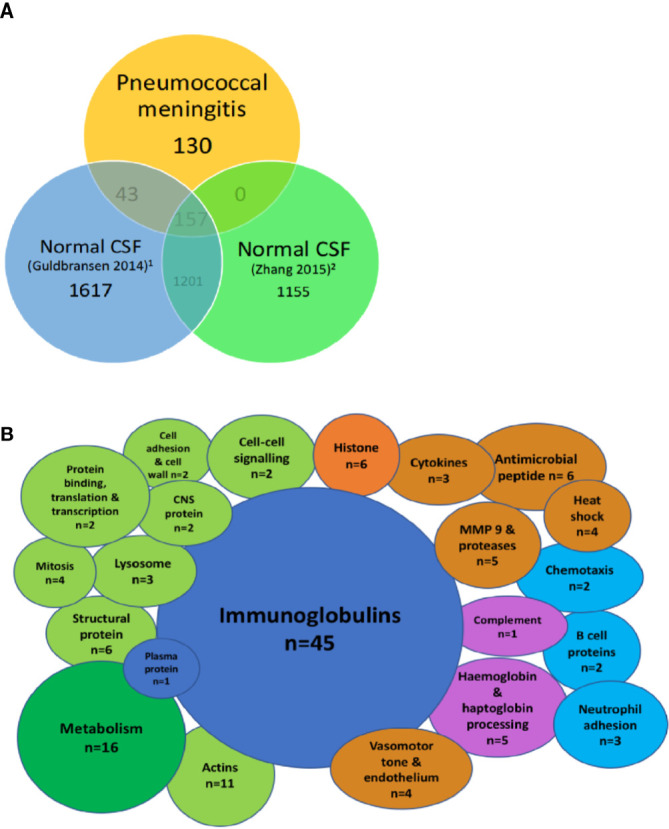
Highly abundant CSF proteins in pneumococcal meningitis are directly involved in the host-pathogen interaction and demonstrate evidence of blood-brain barrier breakdown. Venn diagram showing overlap between the uniport peptide hits from the proteome of patients with PM and two previously published proteomes of normal CSF **(A)**. Bubble plot showing breakdown of proteins found in the subset of proteins unique to PM n = 130 from **Figure 1** **(B)**. Green, cellular functional proteins; blue, proteins synthesized by white and plasma cells; purple, complement and clotting cascades; orange, pro-inflammatory proteins.

When compared to two reports of the proteome in normal CSF (Guldbrandsen et al., 2014; Zhang et al., 2015), we found 130/336 (38%) of proteins in PM were not normally found in CSF (**Figure 2A**). These include the majority of the immunoglobulin sub-classes, neutrophil and inflammatory proteins, CNS proteins, hemoglobin, and some metabolic proteins (**Figure 2B**). Pathways analysis of this protein set unique to PM was strongly enriched for complement activity, hemostasis and heme activation, neutrophil and platelet degranulation, actin dynamics, and scavenger receptor binding (**Table 4**). We also identified 10 proteins with a primary CNS source including *BSAP1*, *APLP1, CHL1*, mapping to pathways including Axon guidance and Dorso-ventricular axis.

**Table 4:** Abundance and function of pneumococcal proteins in the CSF of patients with PM, in order of abundance.

	Protein	Function	Estimated abundance (units)		Protein	Function	Estimated abundance (units)
**1**	Elongation factor Tu	Multi-function	9.20E+05	**11**	Thioredoxin	Stress response	4.46E+04
**2**	Chaperone protein DnaK	Protein folding	2.52E+05	**12**	Enolase	Carbohydrate degradation	4.05E+04
**3**	Ribosomal proteins (n=8)	Ribosome	1.80E+05	**13**	Acyl carrier protein	Lipid metabolism	3.83E+04
**4**	Pyruvate oxidase	Metabolism	1.60E+05	**14**	Endo- Peptidase O	Virulence	2.16E+04
**5**	Ketol-acid reductoisomerase	Metabolism	1.36E+05	**15**	ABC transporter GalT1	Metabolism	1.52E+04
**6**	Glyceraldehyde-3-phosphate dehydrogenase	Metabolism, immuno-stimulant	1.09E+05	**16**	Phosphoglycerate kinase	Metabolism	1.30E+04
**7**	Histone-like DNA-binding protein	DNA stabilization	1.08E+05	**17**	Pyruvate kinase	Metabolism	2.96E+03
**8**	Manganese ABC transporter substrate-binding lipoprotein	Surface antigen	1.03E+05	**18**	Uncharacterized protein	Unknown	2.54E+03
**9**	Glutamate dehydrogenase	Metabolism	4.78E+04	**19**	General stress protein GSP-781	Stress response	2.23E+03
**10**	Elongation factor G	Translation and collagen binding	4.64E+04	**20**	Surface protein pspA	Virulence and surface antigen	2.8E+03

### CSF *Streptococcus pneumoniae* Proteome

Of the 30 pneumococcal proteins detected in CSF, four proteins were structural, ten related to metabolic activity, eight were ribosomal proteins, and nine had primary functions in virulence, or the stress response. These included the virulence factor PspA (involved in avoiding complement mediated immunity), the manganese transporter lipoprotein PsaA (required for protection against oxidative stress), ABC transporter component GalT1 (released during opsonophagocytosis, associated with avoidance of mucosal immunity) (Matthias et al., 2008), and the multi-functional protein Elongation Factor Tu (EF-Tu) (**Table 4**).

### The Human Proteome Did Not Predict Outcome

**Figure 3:**
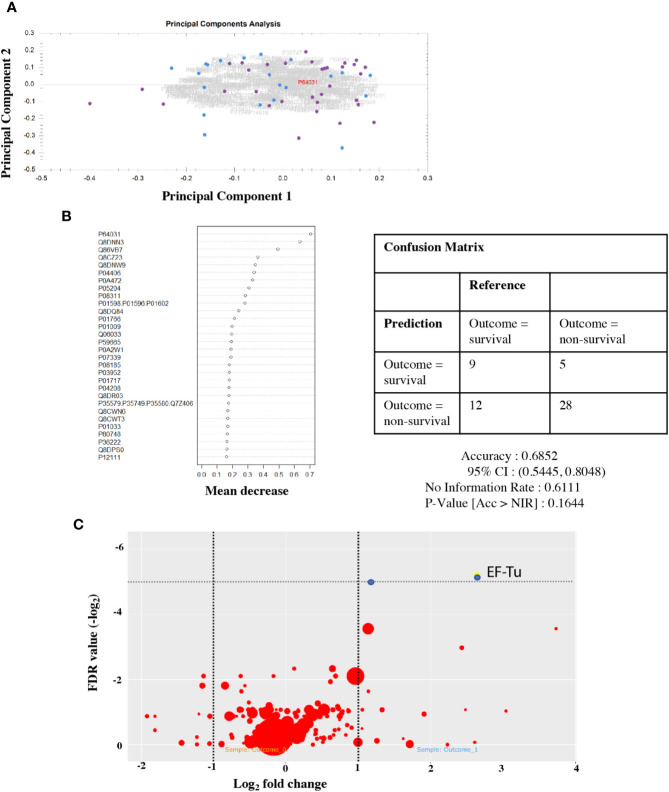
Protein signatures in CSF do not predict outcome from PM, but individual pneumococcal proteins are highly expressed in non-survivors. CSF proteome does not separate by outcome group **(A)**. Principal component analysis of the CSF protein hits. Dot, individual patients; blue, survivor; and purple, non-survivor. Summary plot of Random Forest analysis of highly abundant individual CSF peptide hits **(B)**. Dot per protein shows deviation away from the mean (x axis) toward non-survival. No cluster/signature of abundant proteins accurately predicts outcome in CSF. Quantitative volcano plot of all protein hits **(C)**. Dots represent proteins. Red, non-significantly expressed; blue, significantly expressed (FDR < 0.01). Dot size represents quantitative estimates of abundance.

To investigate the hypothesis that lower GCS reflects worsening inflammation in the CNS, we used a Random Forest model to test for a protein signature to predict outcome in CSF. Principal component analysis of the CSF proteome in PM did not show any separation between outcome groups testing either peptides or proteins (**Figure 3A**). We were unable to detect any sub-set or “signature” of proteins that correlated with outcome status (**Figure 3B**). The Random Forest Accuracy was 0.68 (95% CI 0.54–0.88), no information rate (NIR) 0.61, p-value (Accuracy >NIR) = 0.16. We then tested for associations with individual proteins that may be associated with clinical outcome status (**Figure 3C**). A number of host proteins were over-expressed in the CSF of either patient group (**Supplementary Figure 2**) at an individual level, including S100A8 and CD163. However, none were significantly expressed at >1 log fold change from the mean with FDR <0.05.

### Poor Outcome Was Associated With Higher Levels of the *Streptococcus pneumoniae* Protein Elongation Factor Tu

Although the human CSF proteome did not correlate with outcome in PM, two *S. pneumoniae* proteins identified in the CSF did exceed adjusted FDR significance threshold in non-survivor CSF on the individual association analysis. These were Q8CWR9 (a ribosomal protein) and Elongation Factor Tu (EF-Tu, P64031) (**Figure 3C**). EF-Tu is an immunogenic surface expressed *S. pneumoniae* protein (Thofte et al., 2018; Nagai et al., 2019), involved in the transport of amino acylated tRNA components to ribosomes and is thought to be essential. Recent data suggests that it may also assist *S. pneumoniae* evasion of complement-mediated immunity (Mohan et al., 2014).

### EF-Tu May Inhibit Neutrophil Opsonophagocytosis in Cerebrospinal Fluid

**Figure 4:**
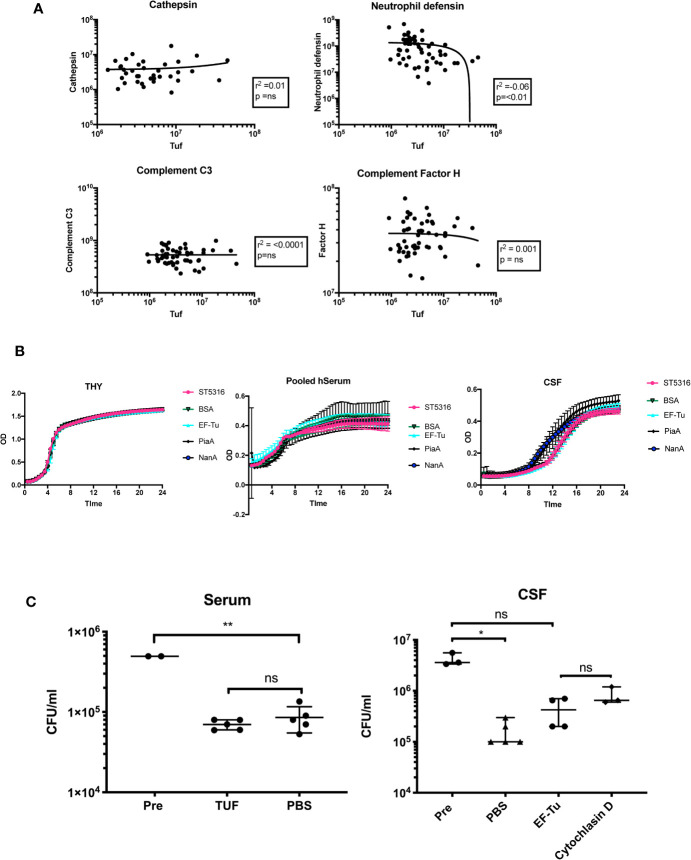
EF-Tu negatively correlates with neutrophil defensin in CSF, and effects on neutrophil-mediated killing in CSF but not serum. **(A)** EF-TU nega**t**ively correlates with neutrophil defensin. Estimates of the abundance of Tuf (x axis, log_10_ scale) plotted against abundance of highly expressed proteins (y axis, log_10_ scale) known to interact with S. pneumoniae (complement C3, Factor H), and the most highly abundant neutrophil protein, Neutrophil defensin **(A)**. Correlation estimated with Spearman’s test. **(B)** EF-Tu does not enhance ST5316 growth in CSF, serum or THY. Growth curves of S. pneumoniae serotype 1 strain ST5316 in Todd-Hewitt broth supplemented with 0.5% yeast extract (THY), pooled human serum and human CSF. Growth plotted over time (x axis) against optical density (y axis) at 620 nm. Growth in normal CSF compared to growth supplemented with 40 ug of recombinant EF-Tu. Additional proteins used as controls, bovine serum albumin (BSA), neuroaminidase A (NanA), and pneumococcal iron acquisition system A (PiaA). **(C)** EF-Tu effects on neutrophil mediated killing of *S. pneumonia*e in CSF but not serum. Viable S. pneumoniae strain ST5316 after 45 min neutrophil opsonophagocytosis assay, supplemented with 400 ug of recombinant Tuf protein. Bacteria opsonized with serum (left panel) and CSF (right panel). Viability measured by colony forming unit (CFU) counts on blood agar after 18 h incubation. Data expressed as medians with range. Statistical significance calculated using the Mann-Whitney U test.

To further investigate the possible effects of EF-Tu on the host-pathogen interaction in PM, we tested for statistical correlations within with host proteins from the 130 unique PM protein dataset. EF-Tu did not positively correlate with any human CSF proteins, but did negatively correlate with levels of neutrophil defensin (r^2^ −0.06, p < 0.01) (**Figure 4A**). Mean CSF levels of EF-Tu in non-survivors were 2.88 log2-fold higher [1.77 × 10^6^ units/ml (Std 1.38 × 10^6^)] compared to 1.64 × 10^5^ units/ml CSF (Std 3.4 × 10^5^) in survivors (ANOVA p −0.05 × 10^5^. In contrast, CSF levels of neutrophil defensin in survivors was 5.76 × 10^7^ (Std 5.61 × 10^7^) compared to 2.4 × 10^7^ (Std 4.2 × 10^7^) in non-survivors (ANOVA p 0.008).

Addition of recombinant EF-Tu did not alter growth of Serotype 1 ST5316 in THY, pooled human serum or hCSF (five technical replicates, three experiments), suggesting no significant effects of EF-Tu on autolysis *in vitro* (Nagai et al., 2018) (**Figure 4B**). Hence, we tested if EF-Tu had a negative effect on the interaction between *S. pneumoniae* and neutrophils in a human CSF model of neutrophil killing. When *S. pneumoniae* were opsonized with pooled human serum, addition of recombinant EF-Tu to the reaction mix did not impair neutrophil killing. However, when bacteria were opsonized with CSF, addition of EF-Tu caused a reduction in *S. pneumoniae* killing compared to PBS control and resulted in a similar effect to inhibition of phagocytosis by cytochalasin D (**Figure 4C**).

## Discussion

The pathogenesis of PM is dominated by a rapid and intense inflammatory response within the CSF compartment driven by an influx of neutrophils reacting to the presence of *S. pneumoniae*. Here, we show the highly inflammatory nature of the proteome in PM on admission to hospital, containing both brain- and blood-derived proteins, but the proteome does not differ between patients who survive and those who subsequently die. Over-expression of bacterial proteins in non-survivors may reflect adaptation of *S. pneumoniae* to the survival in the CSF compartment, that may have negative effects on the innate immune response in PM that requires further investigation.

Human and animal data have shown that the rapid neutrophil influx and the synthesis of antimicrobial peptides triggers the release of highly damaging proteins including tissue proteases (Tuomanen et al., 1985a; Tuomanen et al., 1985b) that in some patients are thought to result in BBB breakdown, cerebral thrombosis, and edema, thereby increasing mortality (Roine et al., 2015; Savonius et al., 2018). We have now replicated our earlier, smaller studies one in children and one in adults of the proteome during PM (Gomez-Baena et al., 2017)^30,31^, using high-throughput quantitative proteomics (Bastos et al., 2017). This method enabled us to estimate abundance of individual proteins, thus determining the most abundant proteins in CSF during PM for the first time (Distler et al., 2016). By adjusting for abundance and using high sensitivity acquisition, we were able to detect all spectra present in CSF and confirm definitively that CSF proteins, whilst highly inflammatory, do not differ between survivors and non-survivors of PM.

Patients in our study were typical for PM patients in Africa, young adults and adolescents, the majority co-infected with HIV, with low CSF WCC and high CSF pneumococcal loads on admission to hospital (Tenforde et al., 2019). As we have previously reported (Wall et al., 2014), neither the bacterial load, CSF WCC and protein concentration, or the HIV status were associated with outcome in our patients. The CSF in patients with proven PM is highly inflammatory. We detected both expansion of the anti-infective components of CSF (immunoglobulins and complement) and high levels of expression of neutrophil-associated proteins including S100A8/9, cathepsin, neutrophil defensin, and matrix-metalloproteinases 9 (MMP9) that infection models have suggested are detrimental to the host (Tuomanen et al., 1989; Roine et al., 2014; Mohanty et al., 2019). Severe BBB breakdown has previously been assumed to be a pre-morbid event in PM (Barichello et al., 2011; Prager et al., 2017), and quantifying BBB breakdown in patients is complex (Helms et al., 2016; Prager et al., 2017; Natarajan et al., 2017). In this dataset, which is controlled for protein abundance, the presence of both brain-derived proteins (e.g. axonal proteins), combined with serum components like hemoglobin, haptoglobin, hemopexin, and fibrinogen, strongly suggest breakdown of the BBB is evident on admission to hospital with PM. We hypothesized that lower GCS on admission was related to worsening BBB breakdown and cerebral inflammation, but this was not supported by the CSF proteome. Our previous study on Malawian adults with PM using unadjusted 2D page proteomics suggested C3 and transferrin were reduced in non-survivor CSF (Goonetilleke et al., 2010; Goonetilleke et al., 2012), and data from The Netherlands also suggested that complement activity in CSF was associated with worse clinical outcomes (Mook-Kanamori et al., 2014; Kasanmoentalib et al., 2015; Kasanmoentalib et al., 2017; Kasanmoentalib et al., 2019). Whilst we demonstrated marked expansion of C3 and transferrin in CSF, after adjusting for overall protein abundance and composition these proteins did not correlate with outcome. These earlier reports were tested only for individual associations between proteins and outcome and were not adjusted for abundance, introducing potential confounding. We also tested if other human CSF proteins singly or in combination using Random Forest models predicted outcome but found no statistically significant association with mortality. The lack of any predictive protein signature suggests that measuring static inflammation through proteins does not reveal the complexity of the host-pathogen interactions associated with tissue damage. Clinical outcomes may instead be associated with dysfunctional processes such as ineffective opsonophagocytosis which are not readily identified by the abundance of specific proteins (Andre et al., 2017; Campos et al., 2017; Ullah et al., 2017; Wright et al., 2017; Lowe et al., 2018; Ritchie et al., 2018).

Compared to the abundance of host proteins, the number of pathogenic proteins identified in our study was relatively small; this probably reflects the relative abundance of bacteria and host cells within the infected CNS. The number and range of *S. pneumoniae* proteins identified in the CSF were similar to our earlier study, including PspA, abundant ribosomal proteins and EF-Tu (Gomez-Baena et al., 2017). These proteins differ from those identified in a CSF proteome study in a murine model of PM (Schmidt et al., 2019), in which high CSF levels of the competence regulator ComDE and the AliB oligopeptide transporter in murine meningitis were associated with leukocyte recruitment to the CSF compartment and disease severity (Schmidt et al., 2019). However, we did not detect either expression of ComDE or AliB in patient PM CSF, and we also did not detect other, previously reported virulence proteins in PM including pneumolysin (Wall et al., 2012; Wippel et al., 2013). The very limited number *S. pneumoniae* proteins detected in this study may be because the abundance of most *S. pneumoniae* proteins were below the limit of detection, proteomics is a relatively insensitive method of assessing bacterial activity during human PM.

Despite the lack of sensitivity of the *S. pneumoniae* CSF proteomics, we found that higher CSF levels of EF-Tu and the ribosomal protein Q8CWR9 were associated with mortality. The over-expression of these proteins in non-survivor CSF is an important finding, and suggests a pathological role during PM. Both proteins are important for *S. pneumoniae* replication, and perhaps therefore reflect rapid bacterial growth as a driver for poorer outcomes. EF-Tu is an essential, highly abundant, ubiquitous bacterial protein, found in diverse prokaryotic species including enterobacteriaceae, *Pseudomonas*, Staphylococci, and *Hemophilus* spp (Harvey et al., 2019). While the primary function of EF-Tu is transport of amino acylated tRNA components to the ribosome, this protein has also been shown to have a surprisingly varied range of moonlighting functions in different bacterial species, including some that affect virulence. EF-Tu can be anchored to the bacterial cell surface, adhere to extracellular components including complement factors (Mohan et al., 2014), chaperone bacterial virulence proteins to the cell surface in membrane vesicles (Olaya-Abril et al., 2014), promote adhesion and invasion of host cells, and alter bacterial shape *via* post-translational modification of bacterial proteins (Harvey et al., 2019). *S. pneumoniae* EF-Tu can bind complement factors H and related proteins Factor HL1 and CFHR1, and plasminogen, has effects on autolysis even provides potent antigen stimulation in an experimental pneumococcal vaccine (Mohan et al., 2014; Nagai et al., 2018; Thofte et al., 2018; Harvey et al., 2019; Nagai et al., 2019). We investigated whether EF-Tu levels in the CSF correlated with complement proteins, proteins associated with BBB breakdown or neutrophil proteins found in the human CSF proteome. Levels of EF-Tu did not correlate with either complement C3 or Factor H (Kasanmoentalib et al., 2019), but did negatively correlate with neutrophil defensin, suggesting a possible interaction with neutrophil opsonophagocytosis in CSF. No correlation was found with other neutrophil proteins in CSF including neutrophil elastase, collagenase, and lipocalin-1.

Neutrophil defensins are multi-functional immunomodulatory proteins with direct anti-microbial activity (Voglis et al., 2009). We tested the hypothesis that excessive EF-Tu effected on neutrophil killing in CSF, finding that EF-Tu promoted bacterial survival when opsonized with CSF but not with serum. The *Pseudomonas aeruginosa* EF-Tu promotes neutrophil apoptosis both *in vitro* and *in vivo* murine pneumonia models an effect that was thought to be mediated by oxygen sensing prolyl hydroxases recognition of EF-Tu (Dickinson et al., 2017). EF-Tu from *S. pneumoniae* may have a similar effect, leading to reduced neutrophil function, especially in the conditions found within CSF compared to serum. Alternatively, functions of EF-Tu may be more effectively attenuated in serum due to components not present in CSF. However, these hypotheses will need more detailed investigation to confirm the association of EF-Tu with mortality and the underlying mechanism(s) involved.

## Limitations

Patients in our study were predominately HIV co-infected, and we were unable to discriminate which inflammatory CSF proteins were directly related to HIV infection rather than PM due to the small number of HIV-negative participants. Neither control CSF nor validated CSF proteomic libraries from healthy individuals living with HIV were available (Wall et al., 2017a). However, data from our center suggests no differences in the transcriptome of children with PM between HIV negative and children living with HIV (Kulohoma et al., 2017). We were unable to stratify HIV co-infected patients by CSF HIV viral load or CD4 count, but all were classified as WHO AIDS Stage 3 with advanced disease. We actively excluded patients with viral co-infection in CSF; infection with hepatitis viruses is common in our setting, and could also potentially through indirect effects on immune function increase the heterogeneity of our host CSF proteomic data. Although we detected an abundance of inflammatory proteins, including those known to be associated with poor outcomes in other studies of PM, we did not detect any cytokines and chemokines known to be present in CSF (Mai et al., 2009; Bociaga-Jasik et al., 2012; Coutinho et al., 2013; Grandgirard et al., 2013; Wall et al., 2014; Roine et al., 2015; Srinivasan et al., 2016). Cytokines and chemokines may be too rapidly degraded to be assessed using mass-spectrometry compared with more sensitive techniques such as ELISA (Kupcova Skalnikova et al., 2017). Visibly traumatic, blood stained CSF was rejected by the laboratory for analysis, however we cannot exclude microscopic blood contamination as a source of CSF hemoglobin/haptoglobin. After extraction of proteins for this study, insufficient CSF remained to validate our findings by measuring CSF levels of EF-Tu by an alternative method. Supporting the proteome data, our unpublished data on gene expression demonstrated high levels of expression of EF-Tu in both patient CSF and during *S. pneumoniae* culture in CSF *in vitro* (unpublished data). Finally, our data are from a single time point, and we cannot determine if dynamic changes in human or *S. pneumoniae* CSF protein predict outcome.

## Conclusions

The CSF proteome in PM is highly inflammatory with evidence of BBB breakdown, but we did not find a human protein signature correlated with clinical outcome. However, higher levels of the *S. pneumoniae* ribosomal protein and EF-Tu were found in non-survivor CSF, and *in vitro* EF-Tu may inhibit neutrophil killing of *S. pneumoniae* in CSF. To better understand the causes of mortality in PM, the role of EF-Tu requires further investigation, using models that accurately reflect conditions in during acute disease.

## Data Availability Statement

The proteomics data presented in this article is publicly available at the PRIDE repository under the accession number PXD021268.

## Author Contributions

EW, GL, PB, VT, RB, AC, SG, RH, and JB conceived and designed the work. EW, GL, VT, MN, VM, TA, ER-S, and BD collected the data. EW, PB, BW, JB, RH, and VT analyzed and interpreted the data. EW drafted the article. EW, PB, GL, VT, VM, BD, MN, TA, ER-S, EC, AC, SG, DL, BW, RB, RH, and JB critically revised the article. EW, PB, GL, VT, VM, BD, MN, TA, ER-S, EC, AC, BG, DL, BW, RB, RH, and JB approved the final version to be published. All authors contributed to the article and approved the submitted version.

## Funding

This study was funded by a Clinical Lecturer Starter Grant from the Academy of Medical Sciences (UK), a UCL-Institutional Strategic Support Fund award to EW from both the Wellcome Trust and the Robin Weiss Foundation and by a PhD Fellowship in Global Health to EW from the Wellcome Trust (089671/B/09/Z). The Malawi-Liverpool-Wellcome Trust Clinical Research Programme is supported by a core grant from the Wellcome Trust (101113/Z/13/Z). The laboratory work was undertaken at LSTM and UCL Respiratory, which received a proportion of funding from the National Institute for Health Research University College London Hospitals Department of Health’s NIHR Biomedical Research Centre. JB is supported by the Centre’s funding scheme. The funders of the study had no role in study design, data collection, data analysis, data interpretation, or writing of the report. The corresponding author had full access to all the data and the final responsibility to submit for publication. JB, VT and BW acknowledge funding from Medical Research Council (MR/R00/1871/1).

## Conflict of Interest

The authors declare that the research was conducted in the absence of any commercial or financial relationships that could be construed as a potential conflict of interest.

## Supplementary Material

Supplementary Figure 1 Confirmation of expression of recombinant pQE30 EF-Tu from *E. coli* 2 and 24 h after protein induction. **(A)** Image of a Ponceau Western membrane showing the presence of pQE30_EF-Tu. *E. coli* containing an pQE30 tagged EF_Tu plasmid. **(B)** Image of a Coomassie stained Western membrane showing the same. Data show experiments in parallel from induced, and non-induced controls. From left to right Ladder, with 55 and 35 KD points indicated respectively. Lane 1 Non-induced isolates, prior to induction. Lane 2 Induced isolates, prior to induction. Lane 3 non-induced isolates, 2 h post induction, Lane 4 induced isolates, 2 h post induction. Lane 5 non-induced isolates, 24 h post induction, Lane 6 induced isolates, 24 h after induction shows the presence of a large 50 kD protein with the pQE30 tag.

Supplementary Figure 2 Individual proteins are more highly expressed in non-survivor CSF. Distribution (estimates of abundance, y-axis, arbitrary units) of proteins individually more abundant (p < 0.05) in non-survivors (purple) compared to survivors (blue), that do not reach significance at the FDR level. Each bar represents an individual patient. Colors represent individual proteins statistically over-expressed in non-survivors. Protein identifiers available in the **supplementary appendix**.
