# Supplementary figures and images for: CSF Levels of Elongation Factor Tu Is Associated With Increased Mortality in Malawian Adults With Streptococcus pneumoniae Meningitis
Source: Front Cell Infect Microbiol. 2020 Dec 11;10:603623. doi: 10.3389/fcimb.2020.603623 (PMC7759504; doi:10.3389/fcimb.2020.603623)

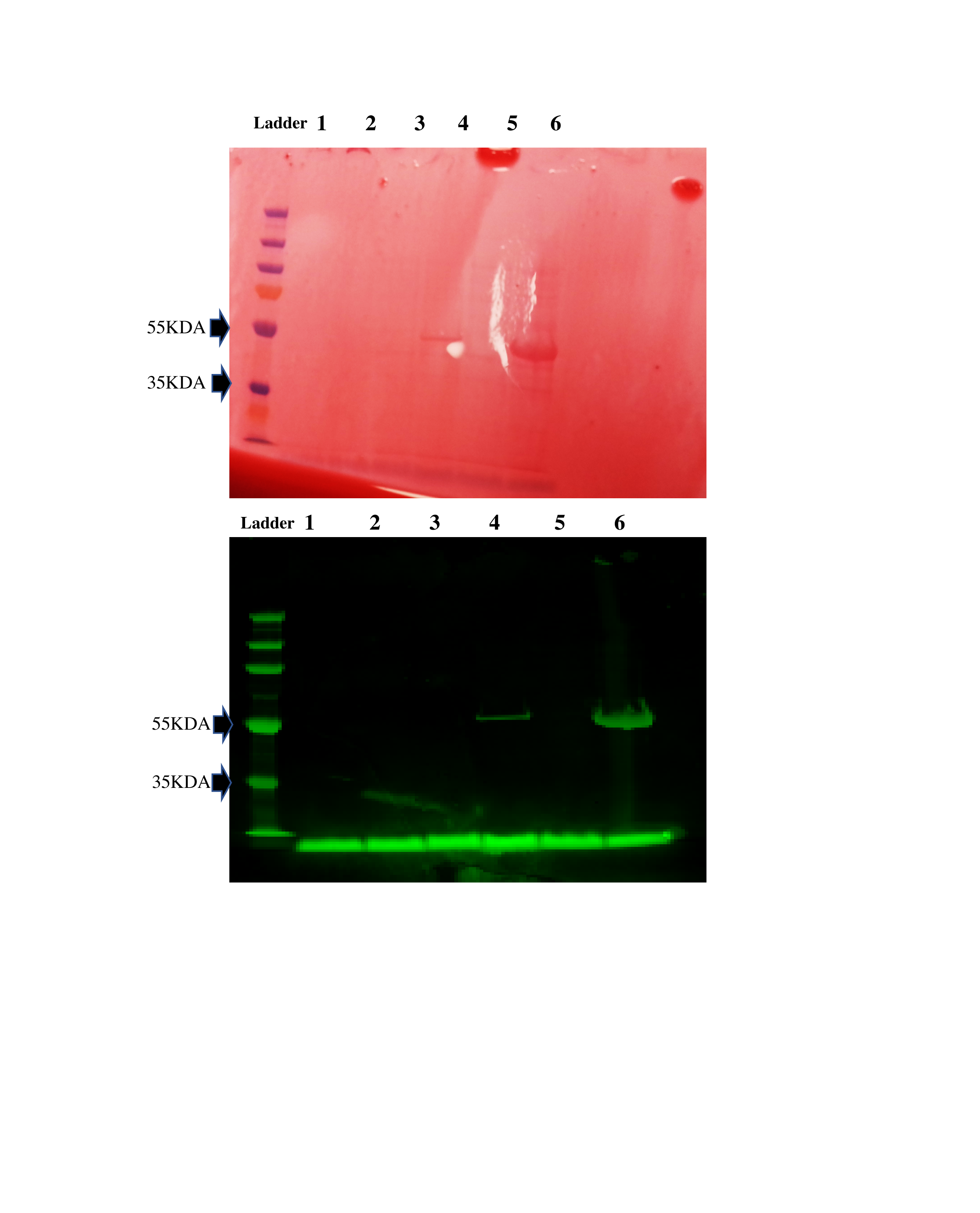

Supplement: Supplementary Figure 1 — Confirmation of expression of recombinant pQE30 EF-Tu from E. coli 2 and 24 h after protein induction. (A) Image of a Ponceau Western membrane showing the presence of pQE30_EF-Tu. E. coli containing an pQE30 tagged EF_Tu plasmid. (B) Image of a Coomassie stained Western membrane showing the same. Data show experiments in parallel from induced, and non-induced controls. From left to right Ladder, with 55 and 35 KD points indicated respectively. Lane 1 Non-induced isolates, prior to induction. Lane 2 Induced isolates, prior to induction. Lane 3 non-induced isolates, 2 h post induction, Lane 4 induced isolates, 2 h post induction. Lane 5 non-induced isolates, 24 h post induction, Lane 6 induced isolates, 24 h after induction shows the presence of a large 50 kD protein with the pQE30 tag. [file Image_1.tif]

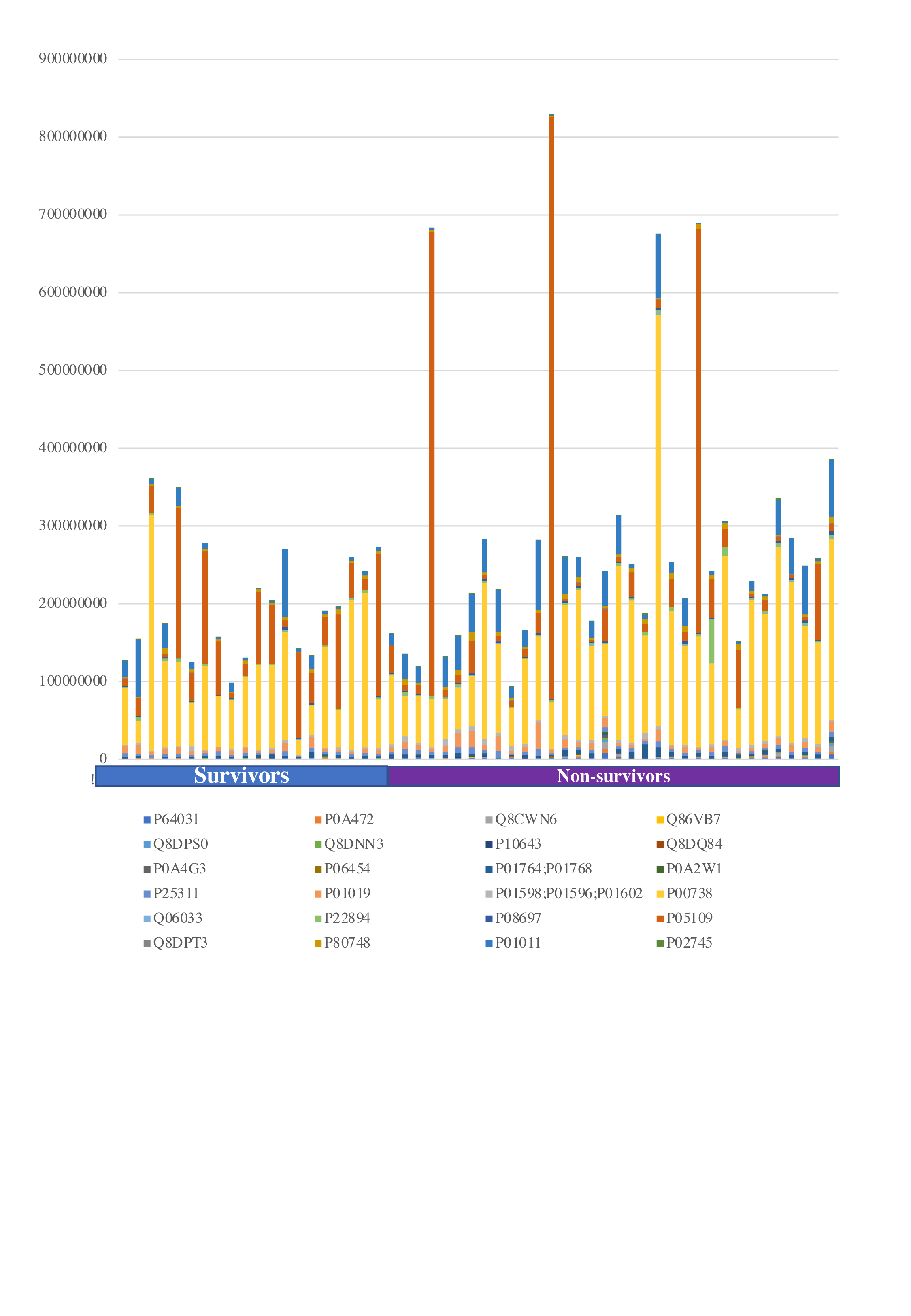

Supplement: Supplementary Figure 2 — Individual proteins are more highly expressed in non-survivor CSF. Distribution (estimates of abundance, y-axis, arbitrary units) of proteins individually more abundant (p < 0.05) in non-survivors (purple) compared to survivors (blue), that do not reach significance at the FDR level. Each bar represents an individual patient. Colors represent individual proteins statistically over-expressed in non-survivors. Protein identifiers available in the supplementary appendix. [file Image_2.tif]
